# Dahongpao mother tree affects soil microbial community and nutrient cycling by increasing rhizosphere soil characteristic metabolite content

**DOI:** 10.3389/fpls.2025.1508622

**Published:** 2025-05-26

**Authors:** Weiting Cheng, Shuqi Zhang, Yuhua Wang, Lei Hong, Miaoen Qiu, Yulin Wang, Yangxin Luo, Qi Zhang, Tingting Wang, Xiaoli Jia, Haibin Wang, Jianghua Ye

**Affiliations:** ^1^ College of Horticulture, Fujian Agriculture and Forestry University, Fuzhou, China; ^2^ College of Tea and Food Science, Wuyi University, Wuyishan, China; ^3^ College of Life Science, Longyan University, Longyan, China

**Keywords:** tea tree, cuttings, soil metabolites, microorganisms, nutrient cycling

## Abstract

Cuttings are an important way of propagating tea trees (*Camellia sinensis*). In this study, Dahongpao mother tree (MD) and cutting Dahongpao (PD) were used as research objects and their rhizosphere soil were collected and performed metabolomics analysis. At the same time, soil nutrient content, microbial physiological indexes, and microbial carbon source utilization were determined, which in turn obtained the effect of cuttings on metabolites, microorganisms, and nutrient cycling in rhizosphere soil of tea trees. The results showed that available nitrogen, available phosphorus and available potassium in the rhizosphere soil of MD were significantly higher (p < 0.05) than in PD. Secondly, microbial biomass carbon, microbial biomass nitrogen, microbial respiration, bacterial number, fungal number, and actinomycete number were also significantly higher in rhizosphere soil of MD than in PD. There were six groups of rhizosphere soil characteristic metabolites that differentiated MD from PD, of which the content of acid, amine, phenol, heterocyclic compound, alcohol and lipid was significantly higher in MD compared to PD, while carbohydrate content was significantly less in MD. There were five groups of rhizosphere soil microorganisms that differentiated MD from PD, in which microorganisms with carboxylic acid, amines, fatty acid and phenolic acid as carbon sources were significantly larger in MD than in PD, whereas microorganisms with carbohydrates as carbon sources were significantly smaller in MD than in PD. It can be seen that the number and content of rhizosphere soil characteristic metabolites were higher in MD than in PD. This enhanced the number of microorganisms with different carbon source utilization rates, increased microbial diversity and abundance, promoted nutrient transformation, increased the content of available nutrients, which in turn facilitated the growth of tea trees. This study provides an important reference for the use of metabolites to regulate soil microbial colonization, improve soil nutrient transformation, and maintain healthy growth of tea trees.

## 1 Introduction

There are numerous methods of artificial asexual propagation of plants, and cuttings are the predominant method of seedling production due to their rapid and efficient characteristics (Megersa, 2017). Cuttings are propagated asexually from mature and good parent plants, whose genomes are theoretically identical to those of the parents and can efficiently inherit the basic characteristics of the parents (Nybom and Lācis, 2021). However, plants from cuttings are susceptible to disturbances from external environmental factors during the growing process, leading to changes in the intensity of gene expression within their different tissues, which in turn affects their metabolic functions (Gil et al., 2020). Second, from the point of view of growth and resistance, even if parents and cuttings are planted in the same environment, there are significant differences in both age and growth, which in turn lead to significant differences in growth rate and resistance strength (Hewitt, 2020; Xiao et al., 2023). It can be seen that although plant cuttings are asexually propagated, there are still some differences between them and their parents, which in turn may affect their growth.

Soil is the medium in which plants are grown, and the rhizosphere is the area in which plants are in close contact with the soil. Changes in the community structure and function of rhizosphere soil microorganisms directly affect the rhizosphere micro-ecosystem, which in turn affects plant growth (Zhao et al., 2021). Singh (2021) found that rhizosphere soil microbial diversity remained significantly different when parent plants and their asexually propagated cuttings were planted in the same plot and managed in the same way. Wang et al. (2024a) subjected *Gastrodia* to multi-generation asexual propagation and analyzed the effect of multi-generation asexually propagated cuttings on soil microorganisms and found that the microbial diversity of the rhizosphere soil of *Gastrodia* declined significantly with the increase in the number of generations of propagation, and the microbial community structure underwent a significant change, which was manifested by the gradual decrease of probiotic bacteria and the continuous increase of pathogenic bacteria. Lin et al. (2024) investigated microbial changes in the rhizosphere soil after asexually propagated sugarcane planting and found significant changes in rhizosphere soil microbial functions, particularly a significant decrease in microbes related to nutrient transformation, which in turn led to a decrease in soil enzyme activities and a reduction in soil nutrient biotransformation. Adomako et al. (2022) explored the rhizosphere soil nutrient transformation capacity of asexually propagated *Solidago canadensis* and found that the nitrogen-phosphorus ratio of the rhizosphere soil of the asexually propagated cuttings was significantly altered, soil nutrient ratios were imbalanced, and the productivity of *Solidago canadensis* was significantly reduced, as compared to the parent. It can be seen that asexually propagated cuttings, although inheriting the characteristics of the parent, have undergone changes in their rhizosphere soil microbial diversity and function during planting, which in turn may have affected the biotransformation of soil nutrients and plant growth.

Tea tree is an important economic plant, and Dahongpao mother tree (*Camellia sinensis*) is an icon of the tea industry in Wuyishan City, Fujian, China, and has been listed by the local government as a key protected object (Chen, 2015). In the mid-1980s, a batch of cuttings were successfully produced by asexual propagation of cuttings for the first time using Dahongpao mother tree as the mother tree (Hou and Wu, 2019). From 2006 to now, in order to effectively protect Dahongpao mother tree, the local government has banned its harvesting for tea production (Ng et al., 2018). As a result, most Dahongpao tea sold on the market today is processed from tea leaves from cuttings of Dahongpao mother tree that were raised into tea trees. Ye et al. (2024) analyzed the quality of Dahongpao mother tree and cutting Dahongpao and found that compared to the parent plant, cuttings had significantly lower aromas such as floral, fruity, green and woody, and significantly lower taste characteristics such as fresh and brisk taste and mellowness. Jia et al. (2024b) analyzed the rhizosphere soil microbial diversity of Dahongpao mother tree and their cuttings and found that the rhizosphere soil microbial diversity of cuttings was significantly reduced, and function was reduced, which in turn affected nutrient uptake by tea trees, and tea leaf quality was reduced. It has been reported that the accumulation of root secretions in rhizosphere soil during plant cultivation is highly susceptible to altering the structure and function of soil microbial communities, which in turn affects soil nutrient transformations and influences plant growth and quality (Shen and Lin, 2021; Tan et al., 2022). The type and content of rhizosphere soil metabolites significantly influence the number and function of soil microorganisms that are addicted to different types of metabolites (Withers et al., 2020). Soil metabolites regulate changes in microbial function, thus affecting soil nutrient transformation and plant growth (Brown et al., 2021; Sun et al., 2022; Wu et al., 2022). It is hypothesized that the changes in growth and quality of cutting Dahongpao may be related to changes in the type or quantity of their rhizosphere soil metabolites. It is of great significance to deeply reveal the metabolite differences between Dahongpao mother trees and cutting Dahongpao, and to search for characteristic metabolites and their effects on soil microbes and nutrient cycling, in order to utilize the metabolites to regulate the reproduction of soil microbes, to regulate the transformation of soil nutrients, and to maintain the healthy growth of tea trees.

Accordingly, in this study, Dahongpao mother tree (MD) and the first asexually propagated cuttings of Dahongpao (PD) in the 1980s were used as objects, and their rhizosphere soils were collected to determine available nutrient content, microbial number, and physiological indexes. Carbon source utilization by rhizosphere soil microorganisms of MD and PD was determined using BIOLOG ECO microplate method. At the same time, metabolomics technology was used to determine rhizosphere soil metabolite compositions and their contents of MD and PD, and to screen for characteristic metabolites distinguishing MD and PD. On this basis, the interactions between soil characteristic metabolites, microorganisms and soil nutrients were further analyzed, with a view to laying a theoretical foundation for metabolites to regulate soil microbial reproduction, improve soil nutrient transformation, and maintain the healthy growth of tea trees.

## 2 Materials and methods

### 2.1 Collection of experimental samples

The sampling site of this study was located in Jiulongke Scenic Area (117°57′19.098″ E, 27°40′17.8212″ N), Wuyishan, Fujian Province, China. In May 2023, rhizosphere soils were collected from MD and PD with three independent replicates of each sample. Among them, MD is about 390 years old, while PD is about 40 years old, and both tea trees are planted in the same plot at a distance of about 20 m. The soil is red gravelly rock. The average annual temperature of the planting site is about 13°C, the precipitation is above 2000 mm and the relative humidity is up to 85%. The sampling method of tea tree rhizosphere soil was as follows: 2 plants of each tea tree were randomly selected, and the surface soil was shoveled layer by layer until the root system appeared (about 20 cm), and the soil attached to the root system was collected, and thoroughly mixed, which was rhizosphere soil. The collected rhizosphere soil was immediately to the ice box, part of it was air-dried and used for the determination of available nitrogen, phosphorus and potassium content, and part of it was used for the determination of microbial population, microbial physiological indexes, microbial carbon source utilization and soil metabolites, with three independent replicates for each sample.

### 2.2 Determination of soil available nitrogen, phosphorus and potassium content

The available nitrogen, phosphorus and potassium content of tea tree rhizosphere soil was determined by Wang et al. (2024b) with three independent replicates for each sample. Briefly, soil available nitrogen was determined by leaching with NaOH solution, and leachate was titrated with hydrochloric acid and then converted to content. Soil available phosphorus was extracted by NaHCO_3_, the extract was added molybdenum-antimony resistance for color development, and then determined by colorimetric method, finally converted to content. Soil available potassium was extracted using ammonium acetate, and the extract was measured directly by flame photometer and then converted to content.

### 2.3 Determination of physiological indexes of soil microorganisms

Microbial biomass carbon, biomass nitrogen and respiration of tea tree rhizosphere soil were determined with reference to Schnecker et al. (2023) with three independent replicates of each sample. Briefly, microbial biomass carbon and nitrogen were determined by chloroform fumigation extraction, i.e., soil samples were fumigated in chloroform for 24 h, extracted with 1 M KCl, and then determined by a TOC/TN analyzer (TOC-l CPH/CPN, Shimadzu, Kyoto, Japan). Where microbial biomass carbon is calculated as (fumigated organic carbon - unfumigated organic carbon)/0.38 and microbial biomass nitrogen is calculated as (fumigated total nitrogen - unfumigated total nitrogen)/0.54. The intensity of soil microbial respiration was measured by the alkali absorption method in mg CO_2_/kg·h, i.e., it was assessed based on the amount of CO_2_ released per kilogram of soil per hour.

### 2.4 Determination of soil bacteria, fungi and actinomycetes number

Quantitative analysis of bacteria, fungi and actinomycetes in tea tree rhizosphere soil was performed by *q*RT-PCR with three independent replicates per sample, as described in Ye et al. (2023). Briefly, 0.5 g of fresh soil was taken and soil DNA was extracted using the Bio-Fast Soil Genomic DNA (BioFlux, Hangzhou, China) extraction kit, and DNA was purified using the gel recovery kit of TianGen Biotech Co., Ltd.The primers used for bacterial quantification were F27 (5′-AGAGTTTGATCMTGCCTCAG-3′) and R1492 (5′-TACHHYTACCTTGTTACGACTT-3′). The bacterial PCR program was set to 95°C pre-denaturation for 4 min, 94°C for 1 min, 55°C for 1 min, 72°C for 1 min, and 35 cycles. The primers used for fungal quantification were 5.8S (5′-CGCTGCGTTCTTCATCG-3′) and ITSIF (5′-CTTGGTCATTTAGAGGAAGTAA-3′). The fungal PCR program was set to 95°C pre-denaturation for 15 s, 94°C for 35 s, 53°C for 30 s, 72°C for 30 s with 35 cycles. The primers used for actinomycetes quantification were act920f (5′-TACGGCCGCAAGGCTA-3′) and act1200r (5′-TCRTCCCCACCTTCCTCCG-3′). The actinomycetes PCR program was set to pre-denaturation at 95°C for 5 min, 95°C for 15 s, 65°C for 30 s, 72°C for 15 s, and 30 cycles.

### 2.5 Extraction and determination of soil metabolites

Extraction and derivatization of rhizosphere soil metabolites of tea trees were performed (Fu et al., 2022). Fresh soil samples were vacuum freeze-dried and ground to powder with three independent replicates for each sample. 0.5 g of the sample was weighed, and 1 mL of extraction solution (methanol:isopropanol:water in the ratio of 3:3:2, v/v) was added, and after shaking for 3 min at room temperature, the sample was placed in an ice bath and ultrasonicated for 20 min, and centrifuged for 3 min at 12,000 r/min at 4°C, and the supernatant was collected. The supernatant was added with 0.02 mL of internal standard (10 μg/mL), mixed well, blown dry under nitrogen, added with 0.1 mL of methoxamine pyridine solution (0.015 g/mL), oximilized at 37°C for 2 h, then added with 0.1 mL of BSTFA (containing 1% TMCS), and the reaction was carried out at 37°C for 30 min to obtain the derivatization solution. The derivatization solution was diluted to 1 mL and passed through a 0.22 μm organic filter membrane for GC-MS testing.

The GC-MS equipment used for the determination of soil metabolites was an Agilent 8890 + 5977B gas chromatography-mass spectrometry instrument (Agilent, Palo Alto, California, USA), and the chromatographic column used was a DB-5MS (30 m × 0.25 mm × 0.25 μm, J&W Scientific, USA). The parameters of GC-MS were set as follows: carrier gas was helium, injection volume was 1 μL, front inlet mode was 5:1, flow rate was 1.2 mL/min; Oven temperature ramp was held at 40°C for 1 min, raised to 100°C at a rate of 20°C/min, raised to 300°C at a rate of 15°C/min, and held at 300°C for 5 min; Transfer line temperature was 280°C, ion source temperature was 230°C, quad temperature was 150°C, and electron energy was 70 eV. Qualitative and quantitative methods for soil metabolites were performed through selecting 2 ~ 3 qualitative ions and 1 quantitative ion for each compound during the determination. and then compared with the NIST20 mass spectrometry database. A compound is qualitative when the selected ions, net of background, all appear in the mass spectrum and the retention time is consistent with the reference value. On this basis, the compound can be quantified by integrating and correcting ions according to the chosen quantification ion (Yuan et al., 2022).

### 2.6 Determination of microbial carbon source utilization

The BIOLOG ECO microplate has 96 wells containing 31 carbon sources, 3 wells per carbon source, i.e. 3 replicates, and 3 blank control wells. The 31 carbon sources can be categorized into six groups, namely carbohydrate, carboxylic acid, phenolic acid, fatty acid, amines and amino acid, respectively (Li et al., 2024). In this study, the utilization of different carbon sources by rhizosphere soil microorganisms of MD and PD was determined using BIOLOG ECO microplate method with reference to Wang et al. (2023). Briefly, 10 g of fresh rhizosphere soil was taken in a conical flask, 90 mL of sterile saline was added, sealed, shaken, and then placed in a shaker at 120 r/min for 10 min. 5 mL of supernatant was taken and diluted 10-fold with sterile water and left to stand, then 5 mL of supernatant was taken again and diluted 10-fold with sterile water to obtain a dilution of 1:1000 for BIOLOG ECO microplate assay. For the BIOLOG ECO microplate assay, 150μL of dilution was added to each well, and a blank well with an equal volume of sterile water was added as a control. The BIOLOG ECO microplate was incubated in a constant temperature incubator at 28°C, protected from light for 7 days, and then the absorbance was measured at 590 nm. The utilization of different carbon sources by rhizosphere soil microorganisms was expressed as Average well color development (AWCD) per pore. AWCD = [∑(C-R)]/31, where C is the absorbance measured after 7 days of incubation in each well and R is the absorbance of the control well.

### 2.7 Statistical analysis

The data obtained in this study were first performed using Excel 2020 for a preliminary statistical analysis of the raw data. Data variances were analyzed using variance analysis (ANOVA) and paired Student ‘s t-tests. The data were plotted using Rstudio software (version 4.2.3), with box and violin plots produced using the R package gghalves 0.1.4, principal component plots produced using the R package ggbiplot 0.55, and bubble heat maps produced using the R package ggplot2 3.5.1. The R package used for orthogonal partial least squares discrimination analysis (OPLS-DA) model construction for MD and PD is ropls and mixOmics, the R package used for bubble feature maps is ggplot2 3.4.0, the R package used for TOPSIS is dplyr 1.1.4, and vegan 2.6.4 for redundancy analysis. The R package used for constructing partial least squares structural squation modeling (PLS-SEM) equations for the different indexes was plspm 0.4.9, and the R package used for correlation-interaction network maps was linkET 0.0.7.1.

## 3 Results and discussion

### 3.1 Analysis of soil available nutrient content, microbial number and physiological indexes

**Figure 1:**
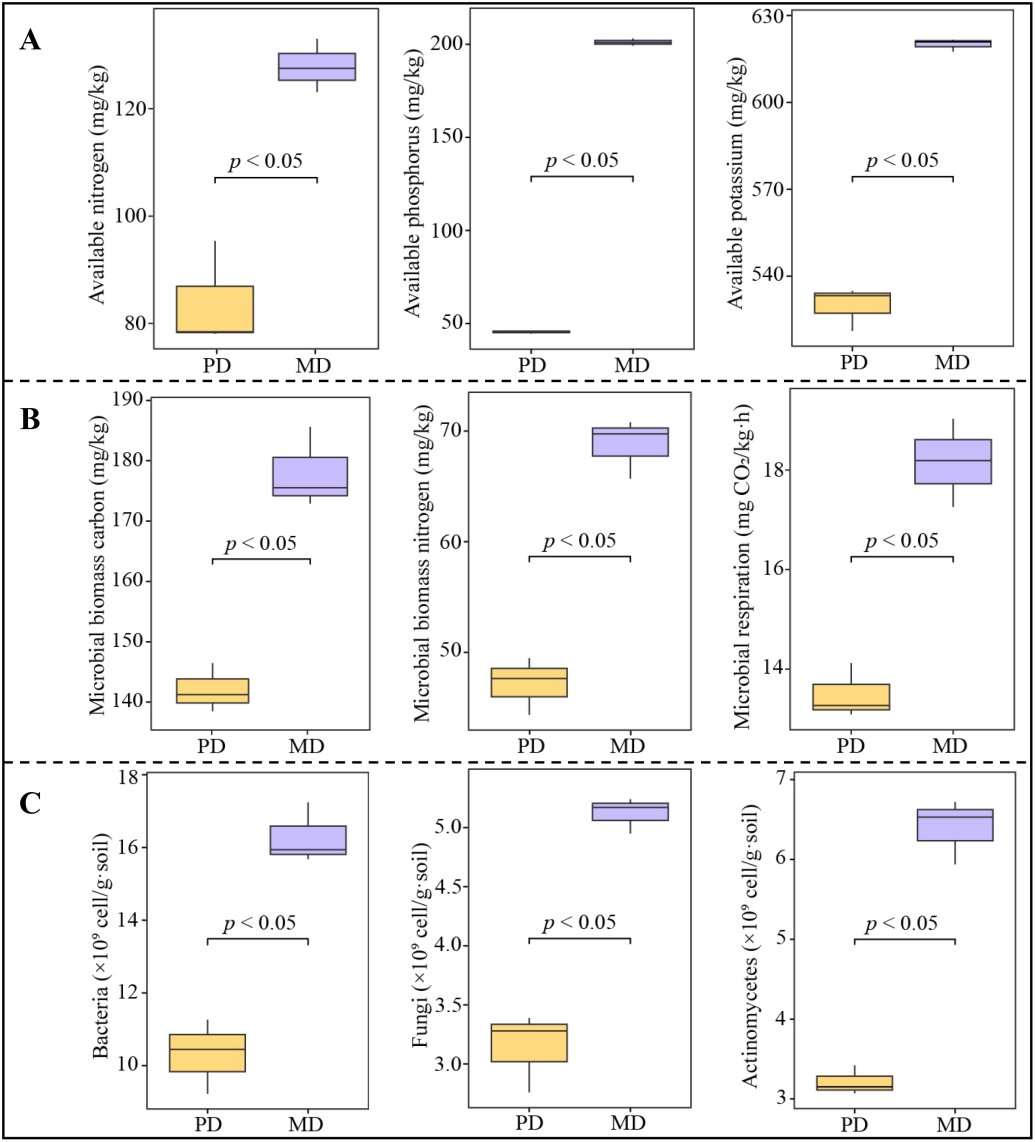
Analysis of available nutrient content and microbial number of rhizosphere soil of tea trees. MD, Dahongpao mother tree; PD, Cutting Dahongpao; **(A)** Analysis of available nutrient content; **(B)** Analysis of microbial physiological indexes; **(C)** Analysis of microbial number.

Plant growth requires nutrient uptake from the soil. Therefore, high or low soil nutrient content, especially available nutrient content, directly or indirectly affects nutrient uptake and accumulation by the plant root system, which influences plant growth (Soares et al., 2019). In this study, available nutrient content of rhizosphere soil of Dahongpao mother tree (MD) and cutting Dahongpao (PD) and found (**Figure 1A**) that available nitrogen, available phosphorus, and available potassium contents of rhizosphere soil of MD were significantly higher than those of PD (*p* < 0.05). Soil available nutrient content is closely related to the biotransformation capacity of soil to nutrients, and the higher the transformation capacity, the more favorable the release of nutrients, which in turn increases the available nutrient content (Macik et al., 2020). The transformation of soil nutrients requires the participation of microorganisms, which can change the soil environment, alter the biotransformation capacity of nutrients, and affect plant growth (Zhong et al., 2020; Bai et al., 2021). In this study, further analysis of rhizosphere soil microbial physiological indexes of MD and PD revealed (**Figure 1B**) that microbial biomass carbon content, microbial biomass nitrogen content, and microbial respiration of MD rhizosphere soil were 178.66 mg/kg, 69.13 mg/kg, and 18.16 mg CO_2_/kg·h, respectively, while those of PD were 142.10 mg/kg, 46.76 mg/kg, and 13.49 mg CO_2_/kg·h, respectively, and MD was significantly larger than PD (*p* < 0.05). Secondly, the analysis of bacterial, fungal and actinomycete numbers showed (**Figure 1C**) that those of MD were all significantly higher (*p* < 0.05) than PD, as evidenced by the fact that the bacterial, fungal and actinomycete numbers in the rhizosphere soil of MD were 16.29×10^9^ cell/g·soil, 5.12×10^9^ cell/g·soil and 6.40×10^9^ cell/g·soil, respectively, while those of PD were 10.32×10^9^ cell/g·soil, 3.14×10^9^ cell/g·soil and 3.21×10^9^ cell/g·soil, respectively. The increase of soil microbial biomass carbon and nitrogen facilitates the mineralization of soil nutrients and improves the biotransformation of soil nutrients (Ma et al., 2020), while microbial respiration characterizes the number of microorganisms in the soil, and the stronger microbial respiration is, the higher the number of microorganisms is, and the more diverse the microorganisms are (Chen et al., 2021). It can be seen that compared with PD, the number of soil microorganisms and their respiration intensity in the rhizosphere of MD were significantly enhanced, which increased the microbial biomass carbon and nitrogen of the soil, enhanced the mineralization capacity of soil nutrients, and promoted soil nutrient biotransformation, which was more conducive to the improvement of soil available nutrient content, and then promoted the growth of tea trees.

### 3.2 Soil metabolite analysis

**Figure 2:**
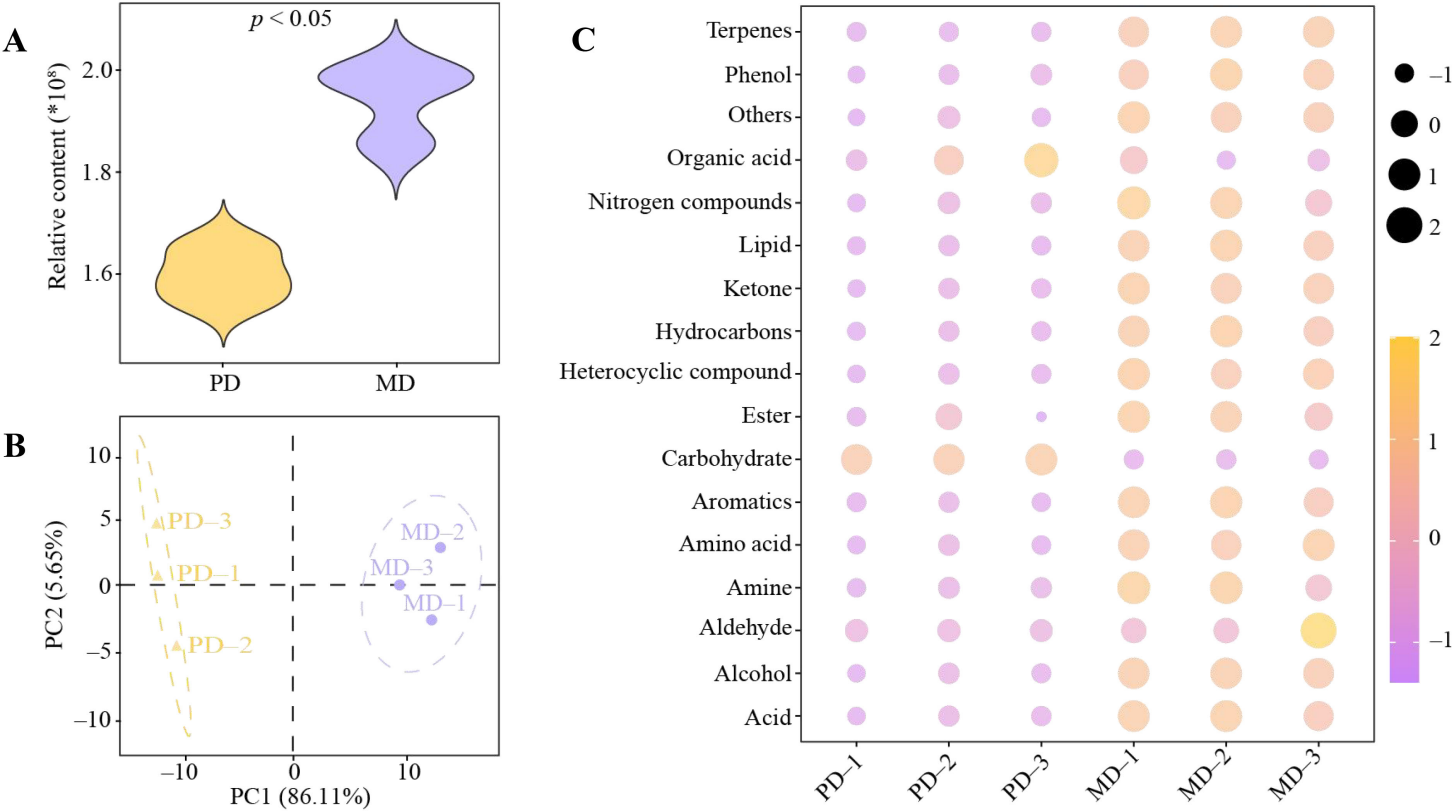
Analysis of metabolite content of rhizosphere soil of tea tree. MD, Dahongpao mother tree; PD, Cutting Dahongpao; **(A)** Total amount analysis of MD and PD rhizosphere soil metabolites; **(B)** PCA analysis of MD and PD rhizosphere soil metabolites; **(C)** Content analysis of MD and PD rhizosphere soil metabolites after classification.

Soil metabolomics can effectively analyze low molecular weight compounds in plant rhizosphere soils and thus assess soil texture (Withers et al., 2020). And, soil metabolomics characterizes the metabolic state of soil biomes and can be used to assess soil function (Wu et al., 2022). Therefore, in this study, soil metabolomics technique was used to analyze the rhizosphere soil metabolites of MD and PD, and the results showed (**Figure 2A**) that the rhizosphere soil metabolite content of MD was significantly higher (*p* < 0.05) than that of PD. PCA analysis showed (**Figure 2B**) that the soil metabolites of MD and PD were significantly different, and the two principal components could effectively differentiate MD from PD with a total contribution of 91.76%. Further analysis of rhizosphere soil metabolites in MD and PD revealed (**Figure 2C**) that a total of 187 metabolites were detected, which can be categorized into 18 groups (including others), of which, 16 groups of metabolites were significantly higher in MD than in PD, namelyterpenes, phenol, nitrogen compounds, lipid, ketone, hydrocarbons, heterocyclic compound, ester, aromatics, amino acid, amine, aldehyde, alcohol, acid and others, whereas 2 groups of metabolite were significantly less in MD than in PD, namely organic acid and carbohydrate. It can be seen that there was a significant difference in rhizosphere soil metabolites between MD and PD, with MD having significantly higher soil metabolite content than PD. Soil metabolite content and types play important roles in the regulation of soil microbial community structure and function (Sikder and Vestergard, 2020). The abundance and diversity of soil metabolites contribute to the diversity of soil microbial communities, which in turn enriches soil microbial functions, enhances soil nutrient biotransformation, and promotes plant growth (Ni et al., 2021; Ortiz and Sansinenea, 2022). It can be seen that the total amount of metabolites and the content of different metabolic types were higher in rhizosphere soil of MD compared with PD, which is more conducive to promoting the proliferation of different types of microorganisms, and more conducive to increasing the richness and diversity of soil microbial community, which in turn promotes the transformation of soil nutrients and influences the growth of tea trees.

### 3.3 Screening and content analysis of soil characteristic metabolites

**Figure 3:**
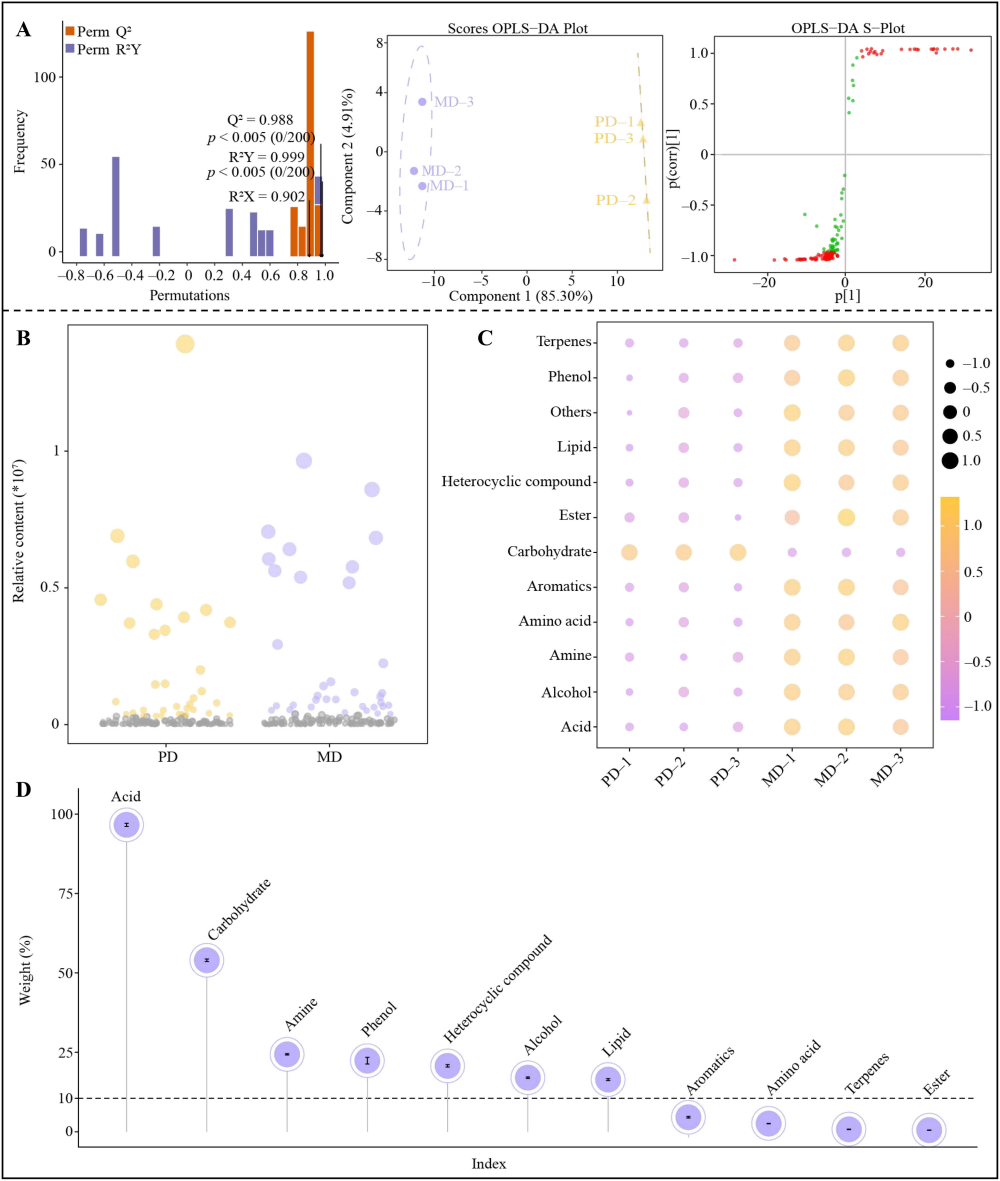
Screening of characteristic metabolites of tea tree rhizosphere soil. MD, Dahongpao mother tree; PD, Cutting Dahongpao; **(A)** Construction of OPLS-DA model for MD and PD and screening for key differential metabolites; **(B)** Screening for characteristic metabolites distinguishing MD from PD by bubble feature map; **(C)** Content analysis after categorization of characteristic metabolites; **(D)** TOPSIS analysis of the contribution of different groups of metabolites in distinguishing MD from PD.

The OPLS-DA model has an important role in screening key metabolites among different samples, which can be used to screen key differential metabolites by the metabolite’s variable importance projection value (VIP) in distinguishing between different samples (Jia et al., 2024a). However, the model needs to be evaluated after construction, and only the model with significant levels of fit and predictability can be used for screening and analysis (Rivera-Pérez et al., 2022; Li et al., 2023b). Accordingly, based on the above analysis, this study constructed an OPLS-DA model of MD and PD with detected soil metabolites and their contents, and screened for key soil metabolites that differentiated MD from PD. The result showed (**Figure 3A**) that the model constructed by MD and PD was tested with a goodness-of-fit R^2^Y value of 0.999 and a predictability Q^2^ value of 0.988, both at significant levels (*p* < 0.05). The OPLS-DA score plot showed (**Figure 3A**) that MD and PD were effectively differentiated, with a difference of 85.30% between groups and only 4.91% within groups. The VIP values of different metabolites in distinguishing MD from PD were obtained by S-Plot plots, and a total of 138 key metabolites with VIP greater than 1 were obtained (**Figure 3A**). The 138 metabolites were further analyzed using bubble feature maps, and 36 characteristic metabolites were screened (**Figure 3B**). Characteristic metabolite categorization analysis showed (**Figure 3C**) that the 36 characteristic metabolites could be classified into 12 groups (including others), of which 11 groups of metabolites were significantly greater in MD than PD, namely terpenes, phenol, lipid, heterocyclic compound, ester, aromatics, amino acid, amine, alcohol, acid and others, while only carbohydrate content in MD was significantly lower than PD. TOPSIS was used to analyze the contribution of each of the 11 groups of metabolites in differentiating MD from PD, and the result showed (**Figure 3D**) that only 7 groups of metabolites contributed more than 10% in differentiating MD from PD, namely acid (96.62%), carbohydrate (53.98%), amine (24.35%), phenol (22.36%), heterocyclic compound (20.72%), alcohol (17.02%) and lipid (16.41%). Tea tree has been reported to be an acidophilic plant with low soil pH, which is suitable for the colonization of acidophilic microorganisms (Yang et al., 2024). Both phenol and alcohol can be converted to acids upon oxidation (De Nobili et al., 2020). Moderate amounts of Amine stimulate nitrogen conversion by soil microorganisms and further convert amine into plant-available and available nitrogen (Paśmionka et al., 2021). Furthermore, when the content of heterocyclic compound in soil is high, it can promote the propagation of bacteria and fungi in soil, increase the efficiency of carbon utilization by microorganisms, enhance the conversion capacity of soil carbon, and promote the growth of plants (Li et al., 2023a). Lipid serves as an important raw material for microbial colonization, which reduces the depletion of available phosphorus in the soil and promotes phosphorus uptake by plants (Warren, 2020). And carbohydrate is an important raw material for microbial reproduction and a major carbon source (Xie et al., 2019). It can be seen that there were significant differences between MD and PD in rhizosphere soil metabolite content, especially characteristic metabolites. The carbohydrate content was significantly higher in the rhizosphere soil of PD compared to MD, which could lead to colonization of rhizosphere soil microorganisms that primarily used carbohydrate as a carbon source. The content of acid, amine, phenol, heterocyclic compound, alcohol and lipid in the rhizosphere soil of MD were significantly higher than that of PD, which could promote the propagation of microorganisms that used different types of carbon sources as raw materials, and was more conducive to increasing the diversity and abundance of the soil microbial community, which was in turn conducive to promoting the transformation of different types of nutrients in the soil.

### 3.4 Microbial carbon source utilization analysis

**Figure 4:**
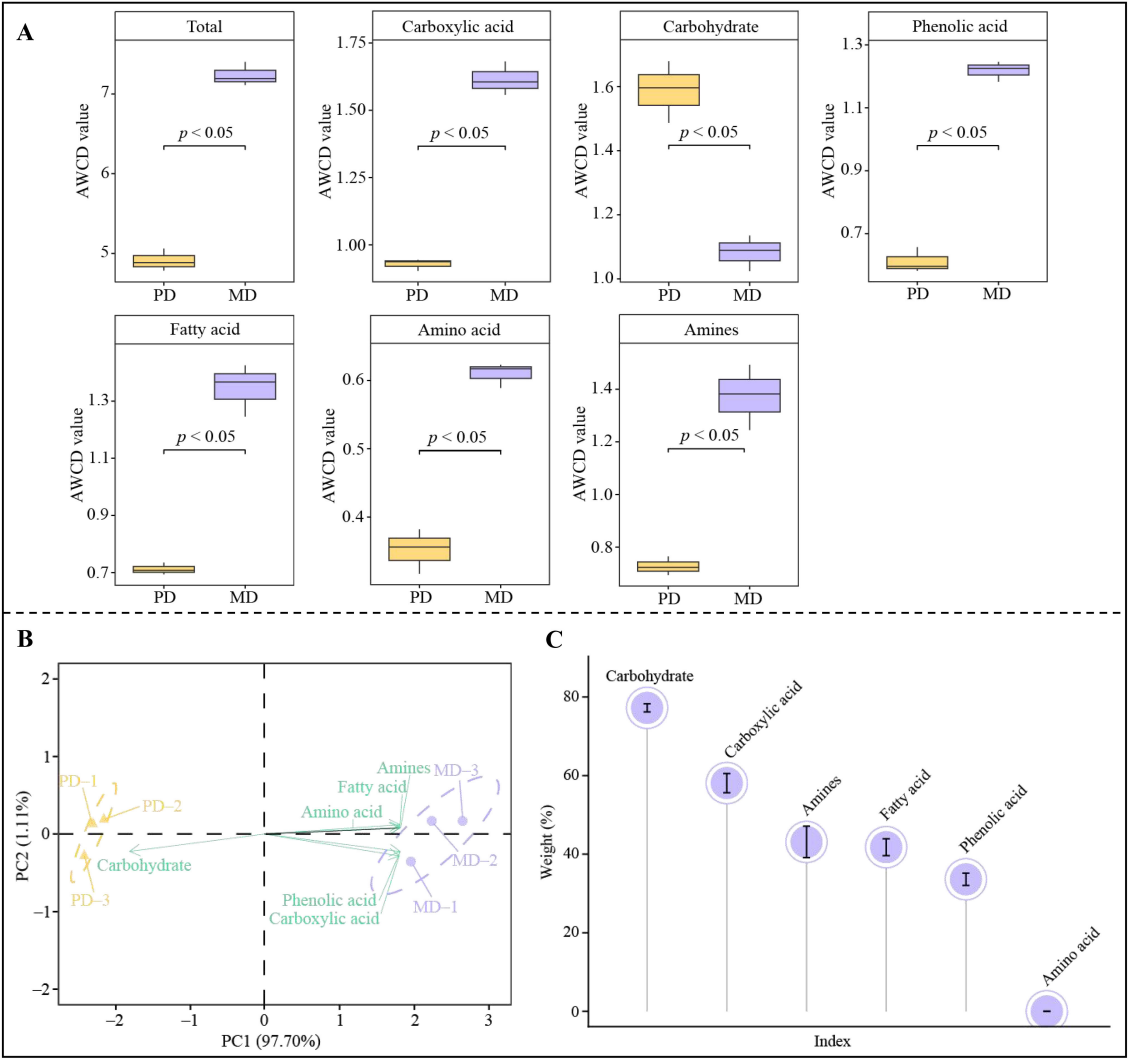
Analysis of microbial carbon source utilization in rhizosphere soil of tea trees. MD, Dahongpao mother tree; PD, Cutting Dahongpao; **(A)** Quantitative analysis of microorganisms utilizing different carbon sources; **(B)** PCA analysis of rhizosphere soils microorganisms utilizing different carbon sources in MD and PD; **(C)** TOPSIS analysis of the contribution rate of microorganisms utilizing different carbon sources in distinguishing MD from PD.

During growth, plants can maintain their own growth by releasing root secretions that regulate the type and amount of rhizosphere soil metabolites in order to adapt to changes in the environment, which in turn affects microbial colonization and alters the structure of the soil microbial community and soil nutrient content (Barra and Terenzi, 2021; Bi et al., 2022). In the present study, significant changes in metabolites were found in rhizosphere soil of MD and PD, which were hypothesized to possibly affect the colonization of the corresponding microorganisms. Accordingly, in this study, the BIOLOG microplate method was used to analyze the number of rhizosphere soil microorganisms utilizing different types of carbon sources in MD and PD, and the results showed (**Figure 4A**) that the overall utilization of carbon sources by rhizosphere soil microorganisms was significantly higher in MD than in PD, and for the utilization of different types of carbon sources, the number of microorganisms using carboxylic acid, phenolic acid, fatty acid, amino acid and amines as carbon sources was significantly higher in MD compared to PD, whereas the number of microorganisms using carbohydrate as carbon source was significantly lower in MD. PCA analysis with different carbon source utilization rates of microorganisms found (**Figure 4B**) that principal component 1 and principal component 2 could effectively differentiate MD from PD, with 97.70% contribution from principal component 1 and 1.11% from principal component 2, giving an overall contribution of 98.81%. And it was found that microorganisms with carboxylic acid, phenolic acid, fatty acid, amino acid and amines as carbon sources were significantly associated with MD, whereas microorganisms with carbohydrates as carbon sources were significantly associated with PD. TOPSIS was further used to analyze the contribution of different microorganisms in distinguishing MD from PD, and the results showed (**Figure 4C**) that only five types of microorganisms that contributed more than 1% to distinguishing MD from PD were microorganisms that used carbohydrate, carboxylic acid, amines, fatty acid and phenolic acid as carbon source, respectively. Phenol can be oxidized to phenolic acid (Rashmi and Negi, 2020), alcohol can be oxidized to carboxylic acid (Pradhan et al., 2020), and lipid can be converted to fatty acid (Dyall et al., 2022). And heterocyclic compound is conducive to promoting soil microbial colonization and enhancing soil nutrient biotransformation (Li et al., 2023a). It can be seen that the content of acid, amine, phenol, heterocyclic compound, alcohol and lipid in soil metabolites was significantly higher in MD than in PD, which in turn was more favorable to promote microbial colonization with carboxylic acid, amines, fatty acid and phenolic acid as carbon sources, whereas PD was more favorable to promote microbial colonization with carbohydrates as carbon sources. The number of microorganisms utilizing different carbon sources in rhizosphere soil of MD was significantly higher than that of PD, and the microbial diversity and abundance of soil was higher, which in turn was more conducive to promoting soil nutrient transformation and tea tree growth.

### 3.5 Interaction analysis

**Figure 5:**
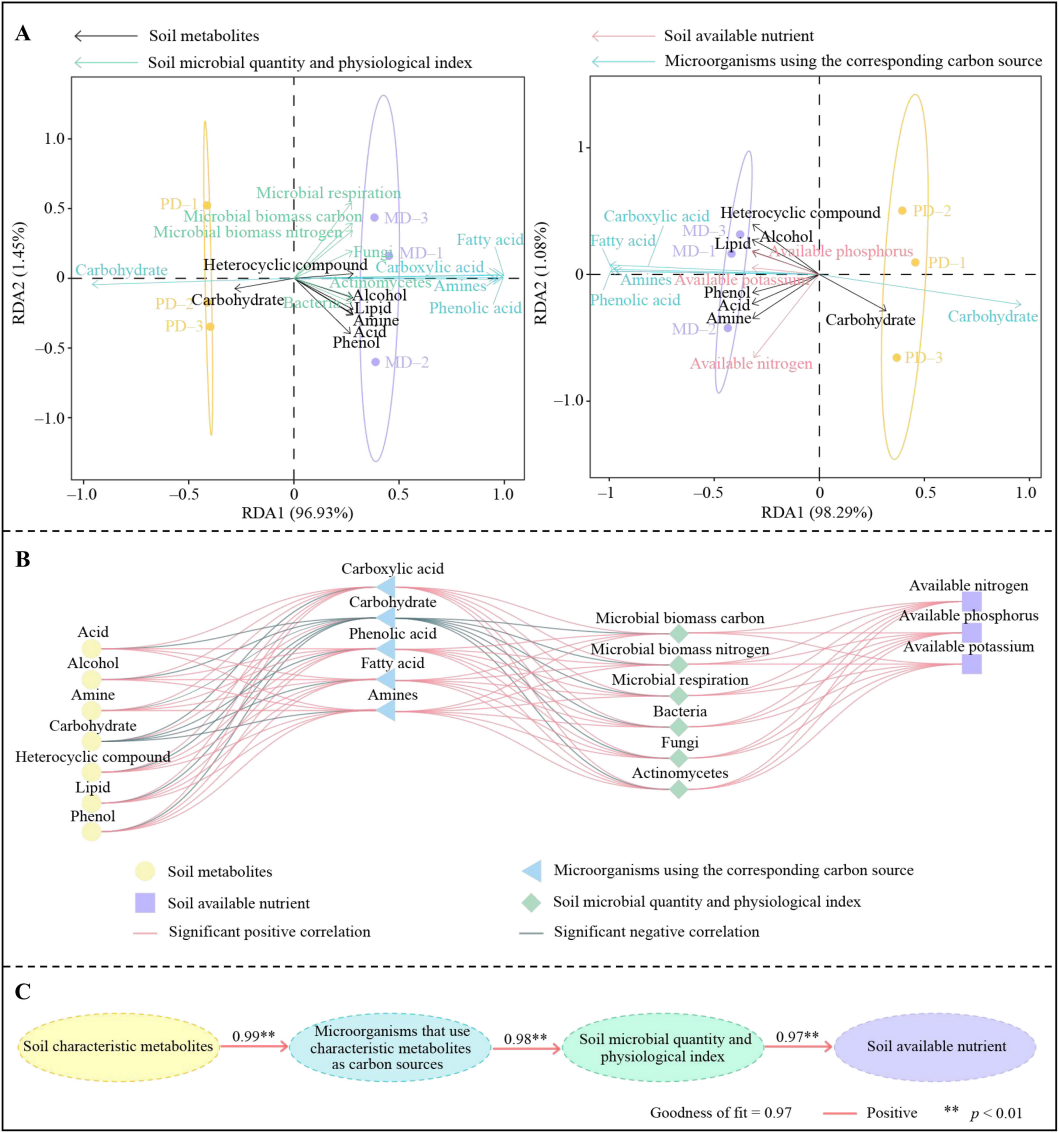
Interaction analysis of soil available nutrients, microorganisms and metabolites. MD, Dahongpao mother tree; PD, Cutting Dahongpao; **(A)** redundancy analysis; **(B)** correlation-interaction network analysis; **(C)** PLS-SEM equation analysis.

In this study, characteristic metabolites in rhizosphere soil of tea trees, microorganisms utilizing different carbon sources were further analyzed for interaction effects with soil available nutrients and microbial physiological indexes. Redundancy analysis showed (**Figure 5A**) that among soil characteristic metabolites, acid, amine, phenol, heterocyclic compound, alcohol and lipid were significantly associated with MD, and soil microorganisms with carboxylic acid, phenolic acid, fatty acid, amino acid and amines as carbon sources were significantly associated with MD. In contrast, both characteristic metabolites and microorganisms utilizing different carbon sources were correlated with soil available nutrient content and microbial physiological indexes. Correlation interaction network analysis showed (**Figure 5B**) that soil characteristic metabolites, microorganisms utilizing different carbon sources, soil available nutrients, and microbial physiological indexes associated with MD were significantly and positively correlated. The PLS-SEM equations of different indexes were further constructed, and the results showed (**Figure 5C**) that rhizosphere soil characteristic metabolites positively regulated microorganisms using different carbon sources in the soil (1.00**), positively regulated soil microbial numbers and physiological indexes (0.98**), and thus positively regulated soil available nutrient content (0.97**). It can be seen that the higher number and content of characteristic metabolites in rhizosphere soil of MD compared to PD was more conducive to increasing the microorganisms using different carbon sources in the soil, which in turn increased the diversity and abundance of soil microorganisms, promoted soil nutrient transformation, improved soil available nutrient content, and promoted tea tree growth.

## 4 Conclusion

**Figure 6:**
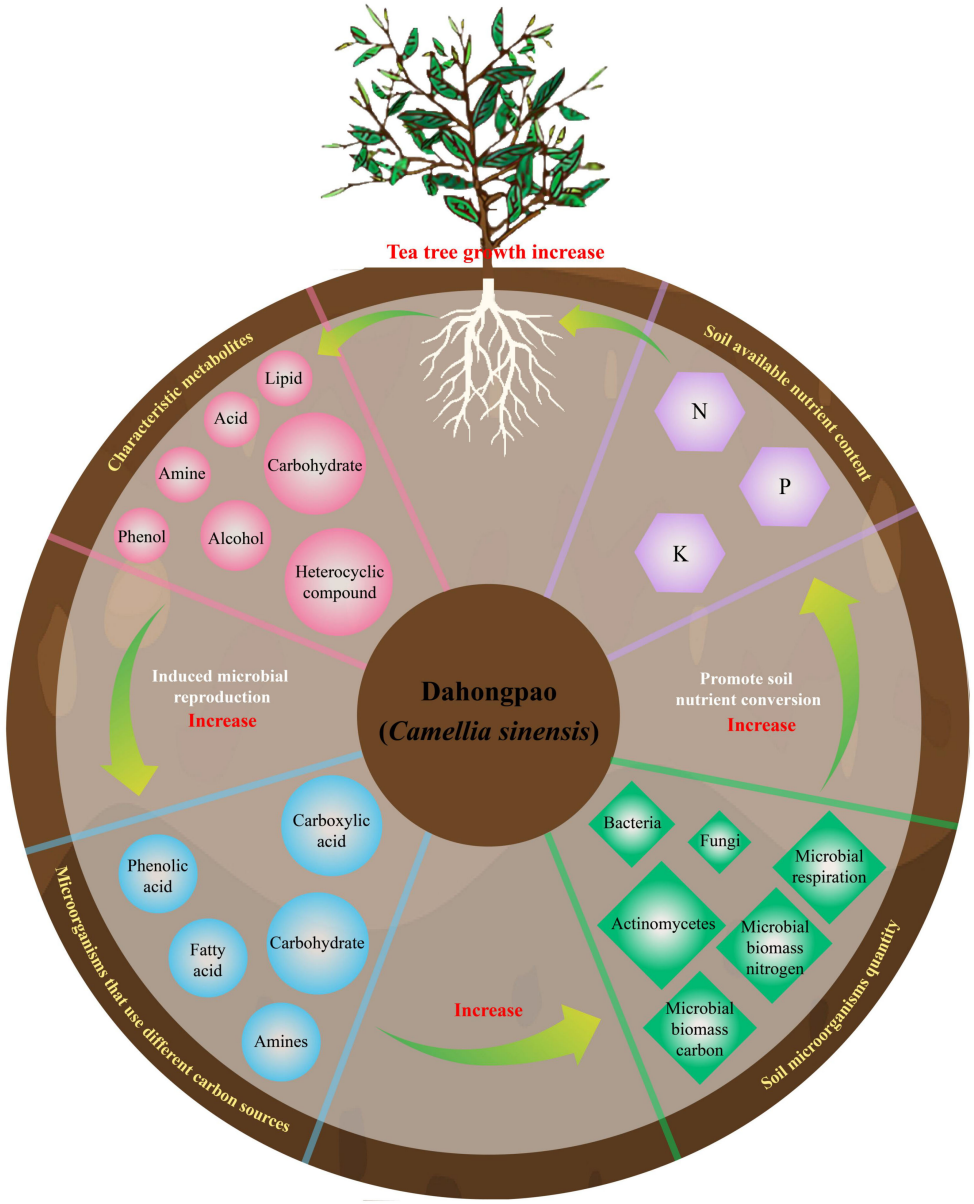
Mechanism analysis of tea rhizosphere soil metabolites regulating soil microbial community affecting soil nutrient cycling.

In this study, Dahongpao mother tree (MD) and cutting Dahongpao (PD) were analyzed for rhizosphere soil available nutrient content, microbial physiological indexes, microbial carbon source utilization and soil metabolites. It was found (**Figure 6**) that MD had significantly higher levels of acid, amine, phenol, heterocyclic compound, alcohol and lipid in rhizosphere soil metabolites than PD, which in turn was more conducive to promoting rhizosphere microbial colonization using carboxylic acid, amines, fatty acid and phenolic acid as carbon sources, whereas PD was more conducive to promoting microbial colonization using carbohydrates as carbon sources. Interaction analysis showed that rhizosphere soil characteristic metabolites positively regulated soil microorganisms using different carbon sources, positively regulated soil microbial numbers and physiological indexes, and thus positively regulated soil available nutrient content. In conclusion, the higher content of characteristic metabolites in the rhizosphere soil of MD compared with PD was more conducive to stimulating the number of microorganisms utilizing different carbon sources, which in turn increased the diversity and abundance of soil microorganisms, and was more conducive to facilitating the transformation of soil nutrients, increasing the content of soil available nutrients, and promoting the growth of tea trees. This study analyzes the effects of cuttings on the rhizosphere soil microecosystem of tea trees from the perspectives of soil metabolites and microorganisms, which is of great guiding significance for the use of metabolites to regulate the propagation of soil microorganisms, improve soil nutrient transformation, and maintain the healthy growth of tea trees.

## Data Availability

The original contributions presented in the study are included in the article/**Supplementary Material**. Further inquiries can be directed to the corresponding authors.

## Supplementary material

Supplementary Table 1 Metabolites in rhizosphere soil of MD and PD.
